# Up‐regulation of SPINT2/HAI‐2 by Azacytidine in bone marrow mesenchymal stromal cells affects leukemic stem cell survival and adhesion

**DOI:** 10.1111/jcmm.14066

**Published:** 2018-11-28

**Authors:** Fernanda Marconi Roversi, Nathalia Moreno Cury, Matheus Rodrigues Lopes, Karla Priscila Ferro, João Agostinho Machado‐Neto, Marisa Claudia Alvarez, Gabriela Pereira dos Santos, Renata Giardini Rosa, Ana Leda Longhini, Adriana da Silva Santos Duarte, Fernando Vieira Pericole, Patricia Favaro, José Andres Yunes, Sara Teresinha Olalla Saad

**Affiliations:** ^1^ Hematology and Transfusion Medicine Center‐University of Campinas/Hemocentro‐UNICAMP Campinas São Paulo Brazil; ^2^ Centro Infantil de Investigações Hematológicas Dr. Domingos A. Boldrini Campinas São Paulo Brazil; ^3^ Present address: Universidade São Francisco (USF) Bragança Paulista São Paulo Brazil; ^4^ Present address: Department of Internal Medicine University of São Paulo at Ribeirão Preto Medical School Ribeirão Preto São Paulo Brazil; ^5^ Present address: Department of Biological Sciences Federal University of São Paulo Diadema Brazil

**Keywords:** de novo acute myeloid leukaemia, mesenchymal stromal cell, methylation, microenvironment niche, myelodysplastic syndromes

## Abstract

The role of tumour microenvironment in neoplasm initiation and malignant evolution has been increasingly recognized. However, the bone marrow mesenchymal stromal cell (BMMSC) contribution to disease progression remains poorly explored. We previously reported that the expression of serine protease inhibitor kunitz‐type2 (SPINT2/HAI‐2), an inhibitor of hepatocyte growth factor (HGF) activation, is significantly lower in BMMSC from myelodysplastic syndromes (MDS) patients compared to healthy donors (HD). Thus, to investigate whether this loss of expression was due to SPINT2/HAI‐2 methylation, BMMSC from MDS and de novo acute myeloid leukaemia (de novo AML) patients were treated with 5‐Azacitidine (Aza), a DNA methyltransferase inhibitor. In MDS‐ and de novo AML‐BMMSC, Aza treatment resulted in a pronounced *SPINT2/HAI‐2* levels up‐regulation. Moreover, Aza treatment of HD‐BMMSC did not improve *SPINT2/HAI‐2* levels. To understand the role of SPINT2/HAI‐2 down‐regulation in BMMSC physiology, SPINT2/HAI‐2 expression was inhibited by lentivirus. SPINT2 underexpression resulted in an increased production of HGF by HS‐5 stromal cells and improved survival of CD34^+^ de novo AML cells. We also observed an increased adhesion of de novo AML hematopoietic cells to SPINT2/HAI‐2 silenced cells. Interestingly, BMMSC isolated from MDS and de novo AML patients had increased expression of the integrins CD49b, CD49d, and CD49e. Thus, SPINT2/HAI‐2 may contribute to functional and morphological abnormalities of the microenvironment niche and to stem/progenitor cancer cell progression. Hence, down‐regulation in *SPINT2/HAI‐2* gene expression, due to methylation in MDS‐BMMSC and de novo AML‐BMMSC, provides novel insights into the pathogenic role of the leukemic bone marrow microenvironment.

## 1 BACKGROUND

*Serine protease inhibitor kunitz‐type 2* (*SPINT2*) codes the HAI‐2 transmembrane protein. The HAI‐2 protein is responsible for the inhibition of the enzyme hepatocyte growth factor activator (HGFA). When active, HGFA is responsible for proteolytic cleavages of the inactive form of the hepatocyte growth factor (pro‐HGF) into HGF active form.^1^ HGF is a multifunctional polypeptide that regulates diverse biological processes such as cell growth, apoptosis, cytokines secretion, adhesion, survival of hematopoietic cells, anti‐inflammation, and immune‐regulation.^2^, ^3^ HGF is secreted by mesenchymal stromal cells (MSC) and the action of this peptide is mediated by c‐met tyrosine kinase pro‐oncogene transmembrane receptor.^4^ Interestingly, HGF cytokine serum levels are significantly increased in myelodysplastic syndromes (MDS) and de novo acute myeloid leukaemia (de novo AML) patients, both heterogeneous diseases, which are prevalent in the elderly, and are a prognostic marker that could predict survival.^5^, ^6^

Low levels of *SPINT2/HAI‐2*, probably due to hypermethylation, have been highlighted in various solid cancer types and have been associated with disease progression.^7^, ^8^, ^9^, ^10^, ^11^, ^12^, ^13^ Pereira and colleagues hypothesized that SPINT2/HAI‐2 is involved in prostate cancer tumourigenesis probably by regulation of SPINT2/HAI‐2.^7^ We previously reported that the expression of *SPINT2* mRNA is significantly lower in bone marrow mesenchymal stromal cell (BMMSC) from MDS patients compared to healthy donors (HD), which could be related with increased HGF and CXCL‐12 secretion.^14^

Despite being related to the pathogenesis of several neoplasms, the role of SPINT2/HAI‐2 has not yet been fully elucidated in haematological malignances, such as MDS and de novo AML. Thus, in this study, we investigate whether this loss of expression was due to SPINT2/HAI‐2 methylation in order to better understand the role of SPINT2/HAI‐2 down‐regulation in MDS and de novo AML physiopathology and its contribution to leukaemic bone marrow microenvironment.

## 2 MATERIALS AND METHODS

### 2.1 Cell

#### 2.1.1 Mesenchymal stromal cell

The BM mononuclear cells were isolated using Ficoll‐Hypaque Plus density‐gradient centrifugation (GE Healthcare). The mononuclear cells were plated onto Dulbecco's modified Eagle's medium (DMEM) (Sigma) supplemented foetal bovine serum (FBS), glutamine, 100 μg/mL penicillin, 100 μg/mL streptomycin, and amphotericin B in a humidified 5% carbon dioxide and 95% air incubator at 37°C. The supernatant with nonadherent cells was removed weekly and replaced with fresh supplemented medium. When the monolayer was established (approximately 90% confluence), cells were trypsinized and plated under the same conditions. After replating them three times, a homogeneous cell population was obtained and MSC were evaluated by flow cytometry for the absence of CD31, CD34, CD45, CD68, and HLA‐DR antigens and the presence of CD73, CD90, and CD105.

#### 2.1.2 HS‐5 stromal cells

HS‐5 stromal cells, representative of human MSCs, were obtained from ATCC. HS‐5 stromal cells were cultured in Roswell Park Memorial Institute medium‐1640 (RPMI) (Sigma) containing 10% FBS, glutamine, 100 μg/mL penicillin, 100 μg/mL streptomycin, and amphotericin B in a humidified 5% carbon dioxide and 95% air incubator at 37°C.

#### 2.1.3 CD34^+^ cells from de novo AML patients

CD34^+^ cells were isolated from BM mononuclear cells by MIDI‐MACS immunoaffinity columns (Miltenyi Biotec) and purity was determined by flow cytometry (minimum of 90%), using anti‐CD34 antibody conjugated to allophycocyanin (APC; Becton Dickinson).

### 2.2 Patients and controls

BM aspirates were collected according to institutional guidelines from healthy donors and from patients with a confirmed diagnosis of MDS and de novo AML, who had attended the outpatient clinic of Hemocentro—UNICAMP from 2005 and 2016 and were untreated at the time of sample collection. BM aspirates were collected from three healthy donors, 10 MDS patients and six de novo AML, classified according to 2008 World Health Organization (WHO). These samples were used to generate primary cultures and to analyse adhesion alpha‐family receptors (CD49, CD49d, and CD49e expressions) by flow cytometry. The ethics committee of the University of Campinas approved this study.

### 2.3 Azacytidine treatment

Mesenchymal stromal feeder layers from 1 HD‐BMMSC, 1 MDS‐BMMSC BMMSC (1 low‐risk MDS‐BMMSC and 1 high‐risk MDS‐BMMSC) and one de novo AML‐BMMSC were seeded into plates (5 × 10^5^ cells/well) in serum‐free RPMI plus bovine serum albumin (BSA) and grown overnight for adherence. Bone marrow mesenchymal stromal cells (BMMSC) were then treated with Azacytidine (AZA, 1 μmol/L) or with vehicle (DMSO) for 48 hours. The cells were then trypsinized, washed, and used to obtain RNA and protein.

### 2.4 Quantitative polymerase chain reaction (qPCR)

Total RNA was extracted from cells using the RNeasy Micro Kit (Qiagen) and cDNA was generated using RevertAid H Minus First Strand cDNA Synthesis Kit (Thermo Fisher Scientific). qRT‐PCR was performed with SYBR Green Master Mix PCR (Thermo Fisher Scientific) using the ABI 7500 Sequence Detection System (Applied‐Biosystem). The relative quantification gene expression values were calculated using the equation 2^−ΔΔCT^ 15 with the housekeeping genes hypoxanthine guanine phosphoribosyltransferase 1 (HPRT1), beta actin (ACTB), and glyceraldehyde‐3‐phosphate desidrogenase (GAPDH). The control was performed for each primer pair. Amplification specificity was verified using a dissociation curve at the end of each run. Three replicas were run on the same plate for each sample.

### 2.5 Western blot

Equal amounts of protein were separated by sodium dodecyl sulphate‐polyacrylamide electrophoresis and transferred to nitrocellulose membranes (Millipore). Detection was carried out with the chemiluminescent substrate using an ECL Plus (GE‐Healthcare). Monoclonal anti‐HGFA Inhibitor 2 (HAI‐2) antibody (ab128926) was purchased from Abcam or anti‐Actin antibody (sc‐1616) from Santa Cruz Biotechnology. Quantitative analyses of the optical intensities of protein bands were determined with Un‐Scan‐It Gel 6.1 (Silk Scientific Inc.) and normalized by actin.

### 2.6 Transduction of lentivirus

HS‐5 stromal cells were transduced with lentivirus‐mediated shRNA nonspecific control (sc‐108080, Santa Cruz Biotechnology) or lentivirus‐mediated shRNA targeting *SPINT2* (sc‐39556‐V, Santa Cruz Biotechnology) and namely shControl and shSPINT2 cells, respectively. HS‐5 stromal cells were seeded (1.5 × 10^5^ cells/well), grown overnight, and transduced with lentiviral vectors at multiplicity of infection equal to 1.0 in a minimal volume of medium containing polybrene (6 μg/mL, Sigma‐Aldrich). The transduced cells were selected for 10‐15 days using puromycin (Santa Cruz). Puromycin resistant cells were expanded and analysed for cytokines production and cell to cell adhesion.

### 2.7 HGF secretion

Mesenchymal stromal feeder layers from shControl and shSPINT2 cells were seeded (1 × 10^5^ cells/well) in serum‐free RPMI plus BSA and incubated for 48 hours at 37°C. Culture supernatants from shControl and shSPINT2 cells were evaluated for HGF human cytokines secretion using a HGF Elisa Kit (ab100534, Abcam). The cytokine concentration was determined from the standard curve. Moreover, shControl and shSPINT2 cells were seeded in a bioscaffold (1 × 10^5^ cells/bioscaffold) produced by our laboratory. These bioscaffolds are well‐preserved 3D microenvironment structures obtained from decellularized bovine bone marrow. Bioscaffolds were cultured for 7 days at 37°C. After this period, the cells on the scaffold were used for an immunohistochemistry (IC) analysis that was performed using the biotin‐streptavidin immunoperoxidase method. Briefly, bioscaffolds were fixed in paraformaldehyde 1% for 5 min. Endogenous peroxidase activity was blocked with 1% H_2_O_2_ and 1% BSA for 30 minutes and then incubated with antibody against HGF (sc13087, Santa Cruz Biotechnology) overnight at 4°C. Following primary antibody incubation, cells were rinsed and incubated with biotinylated secondary antibodies using the Vectastain Elite ABC kit (Vector Laboratories) for 2 hours at room temperature, followed by incubation with diaminobenzidine for 6 minutes at room temperature. The cells were counter‐stained with Harris’ hematoxylin and mounted in permanent mounting medium. Stained cells were examined by light microscopy using an Eclipse i80 (Nikon).

### 2.8 Adhesion analysis of coculture of HS‐5 stromal cells with hematopoietic cells

HS‐5 stromal cells feeder layers from shControl and shSPINT2 cells were seeded in plates (3 × 10^4^ cells/well) in serum‐free RPMI plus BSA and incubated for 48 hours at 37°C. After this period, CD34^+^ cells isolated from total bone marrow de novo AML cells were seeded (3 × 10^5^) onto stromal cell monolayers for another 24 hours. Nonadherent cells were removed by gentle aspiration, and the CD34^+^ cells that adhered to HS‐5 stromal cells were collected by gentle pipetting with cold phosphate‐buffered saline (PBS). Cocultured cells were labelled with CD34‐APC antibodies (BD Biosciences), examined by flow cytometry (FACS Calibur) and analysed by FACS Diva software. Cell percentages expressing the CD34 marker were determined out of a total of 10 000 events.

### 2.9 CD34^+^ cells survival after coculture of HS‐5 stromal cells with hematopoietic cells

HS‐5 stromal cell feeder layers from shControl and shSPINT2 cells were seeded onto plates (3 × 10^4^ cells/well) in serum‐free RPMI plus BSA and incubated for 48 hours at 37°C. After this period, total bone marrow de novo AML cells were seeded (3 × 10^5^) onto stromal cell monolayers in serum‐free RPMI plus BSA and incubated for another 48 hours at 37°C. Nondherent cells were carefully collected by gentle aspiration, labelled with CD34‐APC antibodies (BD Biosciences), examined by flow cytometry (FACS Calibur), and analysed by FACS Diva software. Percentages of cells expressing the CD34 marker were determined out of a total of 10 000 events.

### 2.10 Adhesion molecules profile of mesenchymal stromal cells

Mesenchymal stromal feeder layers from primary cultures generated from MDS, de novo AML and HD BM aspirates were seeded onto plates (1 × 10^5^ cells/well) in serum‐free RPMI plus BSA and incubated for 48 hours. After this period, BMMSC were collected, washed, and resuspended in PBS. BMMSC were incubated for 30 minutes with monoclonal antibodies against the following adhesion alpha‐family receptors CD49b, CD49d, and CD49e (BD Biosciences), evaluated by flow cytometer (FACSCalibur) and analysed using the FACS Diva software. A total of 10 000 events were collected per sample. The distribution histogram was used to determine the geometric mean of fluorescence intensity (MFI) for each antibody tested. The positivity degree for each tested surface adhesion receptor was expressed as a numerical MFI of the positively stained cells.

### 2.11 Statistical analysis

Statistical analysis was performed using GraphPad Prism5 software. Data were expressed as the median [minimum‐maximum]. For comparisons, an appropriate Mann‐Whitney or ANOVA or *t* Student test was used. The values of *P *<* *0.05 were considered as statistically significant. All experiments were repeated at least three times independently.

## 3 RESULTS

### 3.1 Increased *SPINT2* gene and HAI‐2 protein expression after MDS‐BMMSC and AML‐BMMSC treatment with AZA

**Figure 1:**
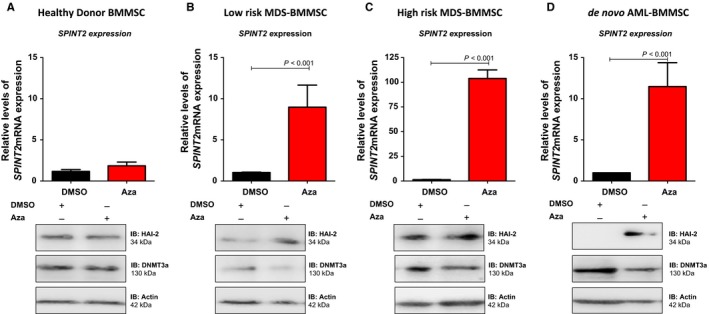
*SPINT2/HAI‐2* expression in bone marrow mesenchymal stromal cells (BMMSC) from patients after Azacytidine treatment. (A) SPINT2/HAI‐2 mRNA and protein expressions in BMMSC from 1 healthy donor treated with Aza for 48 hours. (B) SPINT2/HAI‐2 mRNA and protein expressions in BMMSC from 1 low‐risk myelodysplastic syndromes (MDS) patient treated with Aza for 48 hours. (C) SPINT2/HAI‐2 mRNA and protein expressions in BMMSC from 1 high‐risk MDS patient treated with Aza for 48 hours. (D) SPINT2/HAI‐2mRNA and protein expressions in BMMSC from 1 de novo AML patient treated with Aza for 48 hours. *mRNA* expression levels of *SPINT2* were normalized by *HPRT* endogenous control, as indicated. Results were analysed using 2^−ΔΔ^ ^CT^. Experiments were performed in triplicate. Western blotting was done using protein extracted from total cell. The membrane was blotted with antibodies against SPINT2/HAI‐2 (34 KDa), DMNT3a (130 KDa), or GAPDH (37 kDa), as a control for equal sample loading, and developed with the ECL Western Blotting Analysis System. *P* values are indicated and correspond to the difference between BMMSC treated with vehicle (DMSO) and BMMSC treated with Aza for each sample

As we previously observed a significantly decreased expression of *SPINT2* mRNA in MDS‐BMMSC compared to HD‐BMMSC,^14^ we evaluated *SPINT2* mRNA and HAI‐2 expression in 1 HD‐BMMSC, 1 MDS‐BMMSC (1 low‐risk MDS‐BMMSC and 1 high‐risk MDS‐BMMSC), and 1 de novo AML‐BMMSC, representative sample from each group, after treatment with a demethylating agent, AZA. We initially used HS‐5 cell line as model and treated HS‐5 cell line with AZA or trichostatin. We observed a pronounced up‐regulation of *SPINT2* mRNA expression after AZA treatment for 24 and 48 hours (*P* < 0.05) and no significant alteration in *SPINT2* mRNA expression after trichostatin treatment for 24 and 48 hours (data not shown). Interestingly, AZA treatment for 48 hours in MDS‐BMMSC and de novo AML‐BMMSC resulted in a pronounced up‐regulation of *SPINT2* mRNA and HAI‐2 protein measured by qPCR and Western blot, respectively (Figure 1). SPINT2/HAI‐2 had at least a fivefold increase (between five and seventy‐fold) after AZA exposure in BMMSC from all patients. Moreover, there was no significant difference in *SPINT2* mRNA and HAI‐2 protein expressions between AZA treated and untreated HD‐BMMSC (Figure 1).

We also analysed DMNT3a protein expression, the main target of AZA. As expected, DMNT3a expression was significantly decreased in MDS‐BMMSC and de novo AML‐BMMSC treated with AZA compared to untreated MDS‐BMMSC and de novo AML‐BMMS. No change in DMNT3a expression was observed after AZA treatment of HD‐BMMSC (Figure 1).

### 3.2 Down‐regulation of SPINT2 resulted in an increased HGF secretion, de novo AML CD34^+^ cell adhesion to HS‐5 stromal cells and CD34^+^ de novo AML cells survival

**Figure 2:**
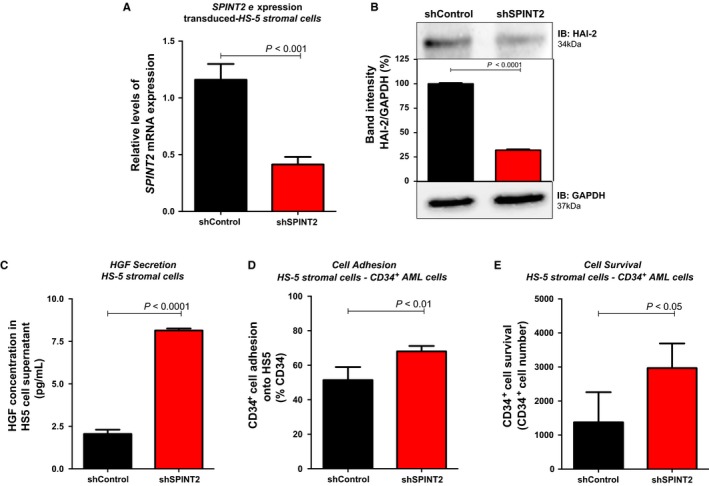
SPINT2 silencing induces HGF secretion, hematopoietic cells adhesion onto HS‐5 stromal cells and CD34^+^ cells survival. (A) Quantitative expression of *SPINT2* mRNA in shSPINT2 cells relative to the shControl cells in HS‐5 stromal cells. Lentivirus‐mediated *SPINT2* shRNA effectively silenced SPINT2 in HS‐5 stromal cells. *mRNA* expression levels of *SPINT2* were normalized by *HPRT* endogenous control, as indicated. Results were analysed using 2^−ΔΔ^ ^CT^. Experiments were performed in triplicate. *P* values are indicated. (B) Western blot analysis of shControl and shSPINT2 total cell extracts of HS‐5 stromal cells. The membrane was blotted with antibodies against SPINT2/HAI‐2 (34 kDa) or actin (42 kDa), as a control for equal sample loading, and developed with the SuperSignal West Pico Chemiluminescent Substrate (Thermo Scientific). (C) Analysis of HGF secreted by shControl and shSPINT2 HS‐5 after 48 hours of culture. (D) CD34^+^ cells from de novo AML patients were added to a monolayer of nontransduced and shControl and shSPINT2 HS‐5 stromal cells and allowed to adhere for 24 hours. After 24 hours, nonadherent cells were removed by gentle aspiration, and the CD34^+^ cells that adhered to HS‐5 stromal cells were collected by gentle pipetting cold phosphate‐buffered saline. The percentage of CD34^+^ adherent cells were measured by flow cytometry using CD34‐APC and analysed as percentage of total cells. Percentages of cells expressing the marker were determined out of a total 10,000 events. (E) CD34^+^ cells from de novo AML total bone marrow were added to a monolayer of nontransduced and shControl and shSPINT2 stromal cells and cultured for 48 hours. After 48 hours, nonadherent cells were carefully collected by gentle aspiration, labelled with CD34‐APC antibodies and measured by flow cytometry. Values are means ± standard deviation of three independent experiments. Statistical analysis: Mann‐Whitney test. *P* values are indicated

To better understand the role of SPINT2/HAI‐2 down‐regulation in MDS‐BMMSC and de novo AML‐BMMSC physiology, we investigated the role of SPINT2 inhibition by transducing human HS‐5 stromal cells with lentivirus‐mediated shRNA targeting SPINT2 or an appropriate control. After puromicin selection, *SPINT2* mRNA and HAI‐2 protein levels were determined by qPCR and Western blot, respectively (Figure 2A,B). Significant decreases in *SPINT2* mRNA (by 65 ± 3%, *P *<* *0.001) and HAI‐2 protein (by 68 ± 4%, *P *<* *0.0001) levels were observed in shSPINT2 cells when compared with shControl cells (Figure 2A,B). These reduced SPINT2/HAI‐2 levels are similar with the decreased *SPINT2/HAI‐2* mRNA expression levels observed in MDS patients when compared with healthy donors (around 61%).^14^

To determine whether alteration of SPINT2/HAI‐2 expression contributes to the production of the HGF cytokine, we measured the levels of HGF cytokines in culture supernatants from shControl and shSPINT2 cells after plating 48 hours without serum induction. Under our culture conditions, an augment in the secretion levels of HGF was observed in shSPINT2 cells when compared to shControl cells measured by Elisa assay (8.20 pg/mL [8.01‐8.22] vs 2.09 pg/mL [1.79‐2.28], *P *<* *0.0001) (Figure 2C).

We also evaluated the influence of decreased SPINT2/HAI‐2 expression in the bone marrow microenvironment, as HGF has been reported to induce adhesion of hematopoietic cells to HS‐5 stromal cells. Thus, we investigated the adhesion between HS‐5 stromal cells silenced or not for the *SPINT2* gene and CD34^+^ de novo AML cells. *SPINT2* gene silencing induced a significant augment in CD34^+^ cell adherence to HS‐5 stromal cells when compared to shControl cells (68.13% [64.33%‐71.49%] vs 52.40% [41.42%‐59.41%], *P *<* *0.01) (Figure 2D).

The next step was to examine the CD34^+^ de novo AML cells growth/survival after a coculture with HS‐5 stromal cells silenced or not for the *SPINT2* gene. A significant increase in CD34^+^ de novo AML cells growth/survival was observed after 48 hours of coculture with shSPINT2 cells when compared to shControl cells (3201 number cells [1969‐3502] vs 983 number cells [841‐2698], *P *<* *0.05) (Figure 2E).

**Figure 3:**
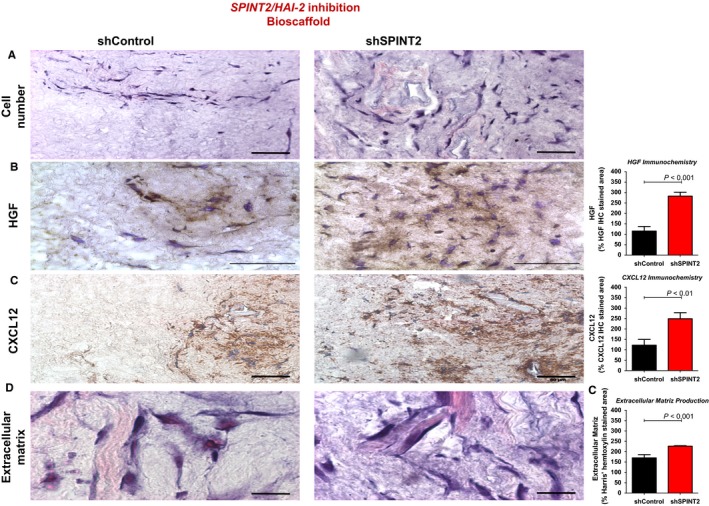
In a bioscaffold (3D tridimensional system culture produced by decellularized bovine bone marrow), SPINT2 silencing induces cell growth, HGF and CXCL12 secretion and matrix extracellular production. Bioscaffold was seeded with shControl or shSPINT bone marrow mesenchymal stromal cell and submitted to (A) Histological staining (40×). (B) HGF immunohistochemistry (100×); (C) CXCL12 immunohistochemistry (100×); (D) Harris’ hematoxylin staining (extracellular matriz production) (100×). The samples were quantified using the National Institutes of Health ImageJ program^32^ and results presented in the graph. Data are the mean ± SEM of different microscopy slide analysis. *P* values are indicated

Moreover, transduced stromal cells were infused into a bioscaffold and were cultured for 7 days. We observed an improvement in cell growth into the bioscaffold containing cells with SPINT2/HAI‐2 depletion (Figure 3A,B). Moreover, our immunohistochemistry analysis showed that the SPINT2/HAI‐2 silenced cell‐seeded scaffold expressed more HGF than the control silenced cells (Figure 3C,D). As HGF is capable of maintaining the stromal microenvironment niche, promoting hematopoiesis by inducing constitutive production of CXCL12, we also analysed CXCL12 expression. Interestingly, the SPINT2/HAI‐2 silenced cell‐seeded scaffold expressed more CXCL12 than the control silenced cells (Figure 3E,F). Since the matrix is important for stem cells adhesion to the stromal cells, we investigated the matrix extracellular production by Harris’ hematoxylin staining. SPINT2/HAI‐2 depleted cells produced more extracellular matrix (ECM) than the control cells (Figure 3G,H).

### 3.3 MDS‐BMMSC and de novo AML‐BMMSC has alteration in the alpha integrin expression profile

The increased CD34^+^ cell adhesion to MSC after SPINT2/HAI‐2 knockdown, prompted us to evaluate the expression of alpha integrins, CD49b (α_2_), CD49d (α_4_), and CD49e (α_5_), in BMMSC from 10 MDS patients, six de novo AML patients, and three healthy donors, as these molecules are known to mediate cell adhesion via HGF.

**Figure 4:**
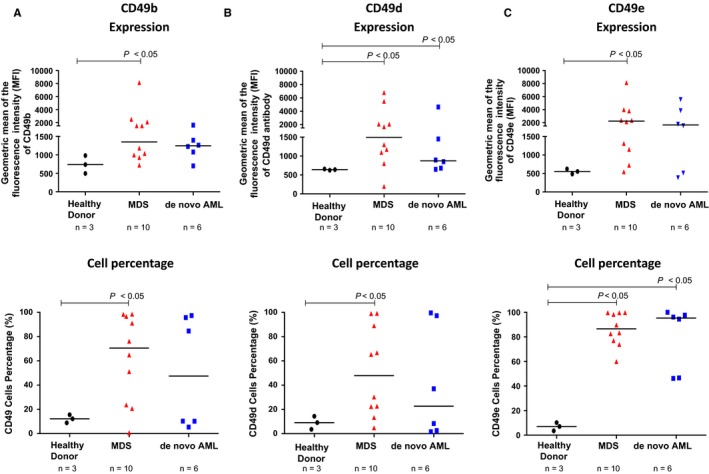
Expression of alpha integrin in bone marrow mesenchymal stromal cell from 3 HD, 10 MDS and six de novo AML patients. The expression of the integrins CD49b and CD49d was analysed by flow cytometry. (A) CD49b expression (MFI) and percentage of total cells expressing CD49b; (B) CD49d expression (MFI) and percentage of total cells expressing CD49d; (C) CD49e expression (MFI) and percentage of total cells expressing CD49e. The distribution histogram was used to determine the geometric MFI. The degree of positivity for each tested surface adhesion receptor was expressed as MFI. Percentages of cells expressing each marker were determined out of a total 10,000 events. Values are means ± standard deviation of three independent experiments. Statistical analysis: Mann‐Whitney test. *P* values are indicated

Compared to HD‐BMMSC, MDS‐BMMSC induced a significant increase in the expression and cell number of CD49b (738.50 [496.0‐981.0] vs 1351.0 [722.0‐8146.0] and 12.20% [8.90%‐15.50%] vs 70.55% [0.80%‐98.50%], *P *<* *0.05), CD49d (642.0 [625.0‐659.0] vs 1495.0 [198.0‐6818.0] and 9.10% [3.60%‐14.40%] vs 47.85% [4.90%‐99.20%] *P *<* *0.05) and CD49e (554.0 [486.0‐622.0] vs 2246.0 [545.0‐8141.0] and 7.10% [3.50%‐10.20%] vs 86.55% [59.90%‐99.80%], *P *<* *0.05), respectively, for HD‐BMMSC and MDS‐BMMSC (Figure 4A‐C). No significant difference in CD49b expression and cell member was observed between HD‐BMMSC and de novo AML‐BMMSC (738.50 [496.0‐981.0] vs 1246.0 [703.0‐1624.0] and 12.20% [8.90%‐15.50%] vs 47.45% [5.40%‐97.40%]), respectively, for HD‐BMMSC and de novo AML‐BMMSC (Figure 4A‐C).

On the other hand, compared to HD‐BMMSC, de novo AML‐BMMSC induced a significant increase in the expression of CD49d (642.0 [625.0‐659.0] vs 875.0 [649.0‐4646.0], *P *<* *0.05) with no significant difference in cell number expressing CD49d (9.10% [3.60%‐14.40%] vs 22.70% [1.60%‐99.60%]). Moreover, de novo AML‐BMMSC induced a significant increase in the cell number expressing CD49e (7.10% [3.50%‐10.20% vs 95.35% [46.20%‐100.00%], *P *<* *0.05), though not in CD49e expression (554.0 [486.0‐622.0] vs 1657.0 [384.0‐5556.0]).

## 4 DISCUSSION

In recent years, the role of tumour microenvironment niche in neoplasm initiation and malignant evolution/progression has been increasingly recognized. Leukemic stem cells are capable of modifying the interaction between hematopoietic stem cells and the bone marrow microenvironment to induce leukemogenesis.^16^ However, the contribution of BMMSC to disease progression remains poorly explored. We had previously performed a microarray analysis of MDS patient‐derived BMMSC (MDS‐BMMSC)^17^ and, after primary MDS and de novo AML BMMSC validation,^14^ found an underexpression of the *SPINT2* gene. This gene encodes a HAI‐2 protein, an endogenous inhibitor of the HGF activator (HFGA), which is responsible for the activation of HGF, a polypeptide secreted by MSC that acts as a multifunctional cytokine, regulating cell adhesion, cell growth, and cell survival of hematopoietic cells through MET pathways.^18^ In MDS and de novo AML patients, the levels of HGF cytokine in the serum are significantly increased and represent a predictor of survival.^5^, ^6^ Kentsis and colleagues showed that up‐regulation of HGF expression was responsible for the maintenance of leukemogenic signalling.^19^

In diverse tumours, the down‐regulation of SPINT2 expression has been associated with gene promoter hypermethylation.^7^, ^8^, ^9^, ^10^, ^11^, ^12^, ^13^ AZA is a DNA methyltransferase inhibitor^20^ that has been successfully used in epigenetic neoplastic therapy to reactivate epigenetically silenced tumour suppressor genes.^21^ Furthermore, AZA is the first‐line treatment for patients with high‐risk myelodysplastic syndrome and has been successful in prolonging survival and delayed AML evolution.^22^ These findings prompted us to examine whether methylation‐mediated epigenetic silencing SPINT2/HAI‐2 was involved in Myelodysplastic Syndrome and de novo AML pathophysiology. Therefore, we obtained primary BMMSCs from healthy donor (HD), MDS, and de novo AML patients, isolated from bone marrow mononuclear cells. HD‐BMMSC, MDS‐BMMSC, and de novo AML‐BMMSC were then treated with AZA. *SPINT2* gene and HAI‐2 protein expressions levels were evaluated by qPCR and Western blot. We observed a significant increase in SPINT2/HAI‐2 levels after AZA treatment in BMMSC from MDS and de novo AML patients, though not in HD‐BMMSC. These results indicate that SPINT2/HAI‐2 is probably epigenetically silenced by methylation in these haematological neoplasms, maybe acting as a tumour suppressor gene in MDS and de novo AML, as occurs in some solid tumours, such as prostate cancer, melanoma, hepatocellular carcinoma, esophageal squamous cell carcinoma, endometrial/uterine cancer, gastric cancer, and medulloblastoma.^7^, ^8^, ^9^, ^10^, ^11^, ^12^, ^13^, ^18^, ^23^

To better understand the role of down‐regulation of SPINT2/HAI‐2 in BMMSC isolated from MDS and de novo AML patients, we inhibited its expression using a lentiviral vector delivering shRNA specific to human *SPINT2* gene, in an HS‐5 stromal cell line immortalized from a bone marrow of healthy donor. To avoid off‐target effects, we used a pool of three shRNAs targeting three different regions on *SPINT2* gene as well as shRNA that target irrelevant genes (negative control). After confirmation of gene and protein SPINT2/HAI‐2 inhibition, cells were grown to analyse HGF secretion. Our results showed that cells transduced with shSPINT2 expressed and secreted more HGF cytokine than shControl cells, probably due to the reduction in the HGFA enzyme inhibition, leading in the cleavage of pro‐HGF to HGF active heterodimer. HGF is capable of maintaining the stromal microenvironment niche by inducing constitutive production of CXCL12. Our analysis has shown an improved CXCL12 expression in cells transduced with shSPINT2. HGF and CXCL12 increased secretions occurred as a consequence of an increased cell proliferation and individual cell secretion.

Mesenchymal stromal cells production of HGF has protective effects on hematopoietic cells due to anti‐inflammatory, anti‐fibrotic, anti‐apoptotic, and cell‐to‐cell adhesion mechanisms.^2^, ^3^ Crosstalk between MSC and hematopoietic stem cells, in the bone marrow microenvironment, are essential to support stem cell behaviour and contribute to abnormal hematopoietic cell growth, survival, and maturation.^24^, ^25^ Moreover, the cell‐to‐cell contact between BMMSC and myelodysplastic and leukemic stem cells is important for cell self‐renewal, proliferation, and survival as well as for leukemogenesis.^26^, ^27^ In this concern, we evaluated the contribution of SPINT2/HAI‐2 silencing in cell‐to‐cell adhesion. After a coculture between MSC and CD34^+^ cells, we observed an increased adherence between shSPINT2 MSCs and hematopoietic stem cells (CD34^+^ cells) isolated from de novo AML patients.

As the interactions and adhesion between MSC and CD34^+^ cells are critical for survival of all cells, mainly for leukemic cells, we also verified an improved survival and growth in CD34^+^ de novo AML cells after coculture with shSPINT2 MSCs. Our results are in accordance with the results of Eksioglu‐Demiralp who showed that HGF has a survival‐protective effect on B‐CLL cells in vitro.^28^ Thus, we hypothesize that lower levels of SPINT2/HAI‐2 induced an increase in HGF secretion, which acts on de novo AML cells through a paracrine loop, stimulating the HGF‐MET pathway and resulting in an improved adhesion of CD34^+^ de novo AML cells to HS‐5 stromal cells and CD34^+^ cell survival, which is an important mechanism of leukaemia chemotherapy resistance.

Amongst several families of adhesion molecules, integrins have a special interest in hematopoietic cells. The alpha integrins are known to play an important role in the interaction between hematopoietic stem cell and bone marrow microenvironment niche, controlling hematopoiesis.^29^ In this sense, we analysed the expression of the alpha integrin family (CD49b [α_2_], CD49d [α_4_] and CD49e [α_5_]) in BMMSC from healthy donors, myelodysplastic syndrome patients, and AML patients. We observed that BMMSC from MDS and de novo AML patients induced a significant increase in the expression and the cell percentage of CD49b (α_2_), CD49d (α_4_), and CD49e (α_5_) proteins. Since CD49d proteins are responsible for CD34^+^ cells or blast attachment and homing to MSC^30^ an increased CD49d expression in MDS‐BMMSC and de novo AML‐BMMSC probably improves attachment, retention, self‐renewal, and homing of hematopoietic and leukaemia cells to the bone marrow niche, modulating chemotherapy response. Moreover, CD49e participates in the activation of some kinases that regulate cell growth, survival, differentiation, and/or migration.^31^ Thus, CD49d probably helps to the maintain CD34^+^ cells in contact with BMMSC in MDS‐BMMSC and de novo AML‐BMMSC, and CD49e probably activates some kinase signalling pathway in leukaemic cells that increase cell survival and proliferation. Furthermore, CD49b and CD49e are essential for the cell attachment to the ECM.^31^ In this way, increased CD49b and CD49e expressions in MDS‐BMMSC and de novo AML‐BMMSC may mediate the adhesion of these BMMSC to the ECM. Therefore, silenced SPINT2 cells produced more ECM than control cells, as we demonstrated using the bioscaffold 3D‐culture assay.

**Figure 5:**
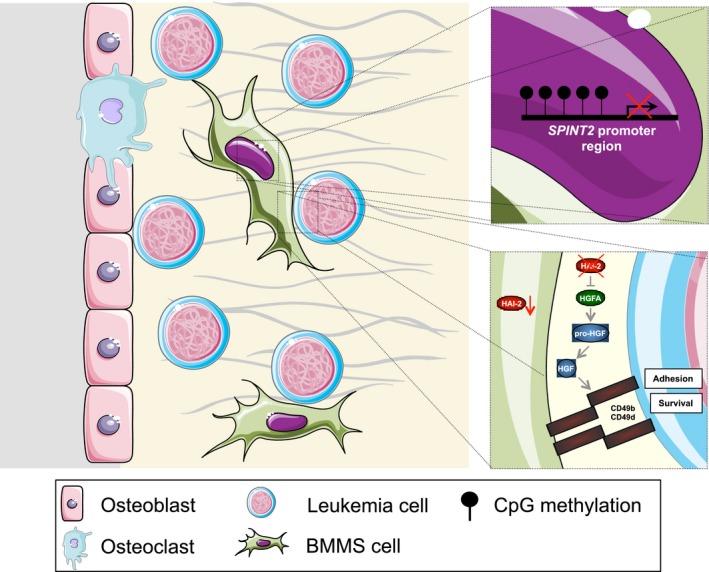
Schematic pathway hypothesis of the biological effects of SPINT2/HAI‐2 in haematological neoplasms (Myelodysplastic Syndrome and Acute Myeloid Leukemia, AML). Methylation results in the inhibition of SPINT2/HAI‐2 expression in bone marrow mesenchymal stromal cells from myelodysplastic syndromes (MDS) and de novo AML patients, resulting in increased secretion of HGF with consequent increase in cell adhesion and in survival/growth of hematopoietic cells, mainly of the abnormal MDS and de novo AML cells, and contributing with the functional abnormalities of microenvironment niche and with cancer progression. This figure was created using Servier Medical Art tools (http://www.servier.com)

Hence, the down‐regulation in SPINT2/HAI‐2 expression profile, probably due to promoter methylation, in MDS and de novo AML BMMSCs, provides novel insights of SPINT2/HAI‐2 into the pathogenic role of the dysplastic and leukaemic bone marrow microenvironment. Likewise, our results suggest that SPINT2/HAI‐2 may play a role in the deregulation of HFG and CXCL12 cytokine secretion with consequent alteration in dysplastic and leukaemic CD34^+^ cell adhesion and growth/survival. Therefore, SPINT2/HAI‐2 may participate in the functional and morphological abnormalities of the microenvironment niche and cancer progression, possibly contributing to chemotherapy resistance. Thus, we hypothesize that SPINT2/HAI‐2 could be a new prognostic marker and an interesting therapeutic target (Figure 5).

## AUTHORS’ CONTRIBUTION

F.M.R. served as the principal investigator for this study, designed and performed the experiments, collected, analysis and interpreted of data, wrote the manuscript; N.M.C., M.R.L., and M.C.A.P. contributed to the experiments, analysed and interpreted data JAMN analysed and interpreted data and helped in the writing of the manuscript; K.P.F. provided technical assistance for the western blot analysis; A.L.L. provided technical assistance for the flow cytometry analysis; A.S.S.D. carried out the cell separation; F.V.P. acted as reference physicians; P.F and J.A.Y. helped analyse the data; S.T.O.S. provided the study conception, directed the research, provided financial support, revised and approved the manuscript. All authors have read, commented, and approved the final version of the manuscript.

## CONFLICT OF INTEREST

No competing financial interests exist.
